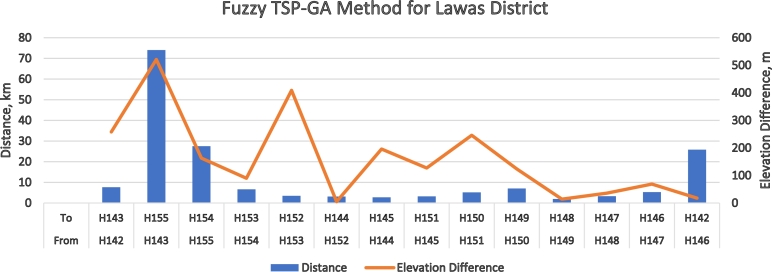# A new hybrid Artificial Intelligence (AI) approach for hydro energy sites selection and integration

**DOI:** 10.1016/j.heliyon.2022.e10638

**Published:** 2022-09-21

**Authors:** F. Chen Jong, Musse Mohamud Ahmed, W. Kin Lau, H. Aik Denis Lee

**Affiliations:** a Department of Electrical and Electronic Engineering, Universiti Malaysia Sarawak, Kota Samarahan, Sarawak, Malaysia; b Department of Grid System, Sarawak Energy Berhad, Kuching, Sarawak, Malaysia

**Keywords:** Fuzzy logic operation, Genetic algorithm, Hydro energy sites, Mixed integer-linear-programming, Travelling salesman problem

## Abstract

The increase of energy demand in this era leads exploration of new renewable energy sites. Renewable energy offers multiple benefits; hence it is suitable to be harnessed to meet power needs. In Sarawak, exploitation of hydro energy is a very feasible potential due to the abundant river flows and high rainfall volume. Thus, in this paper, 155 potential Hydro Energy Sites (HES) are identified and divided into six districts using a raw and unprocessed data provided by Sarawak Energy Berhad (SEB). Since there are no similar researches previously done for identification and integration of hydro energy sources, in this paper, two stage complex data management was built using 155 HES locations in Sarawak. New spatial mapping technique were used for the first stage. From the new spatial mapping technique, the mapped data were categorized into groups, analysed and created new accurate mapping locations on the Sarawak map in terms of the districts using GIS Spatial tools. Their exact geographical locations were identified, and their coordinate systems have been retrieved as complete final data with geo-referencing technique in QGIS with ID numbers. Moreover, the power capacity of each location of all the 155 HES was quantified. By employing this data, the identified locations have been integrated into the already created 155 HES sites. For the second stage, a new two-part AI hybrid approach has been proposed and applied to improve optimal transmission line routing for each district to locate transmission line paths. The first part of hybrid AI implemented in this paper was TSP-GA and second part implemented in this paper was based on improved fuzzy logic with TSP-GA together. To ensure the optimal results are reliably achieved, both first part of TSP-GA and second part of improved fuzzy TSP-GA are utilized to generate the transmission line routing. These two approaches are required to obtain the minimal values of total distance and total elevation difference of each HES. Based on the benchmarking results, fuzzy TSP-GA successfully improved 12.99% for Song district, 7.52% for Kapit district, 3.71% for Belaga district, 1.54% for Marudi district, 18.01% for Limbang district, 11.00% for Lawas district when comparing against the ordinary TSP-GA approach.

## 1 Introduction

Renewable energy is receiving significant attention nowadays. It is because renewable energy possesses clean, omnipresent, and infinite properties. It has also been gradually replacing the usage of fossil fuels [1]. In Sarawak, various types of renewable energy sources are worthy for implementation. Since Sarawak is blessed with high rainfall about 4600 millimetres annually and abundant river flows, harnessing hydro energy is an excellent option due to these local geographical characteristics [2]. In Sarawak, the longest river is in the northwest of Borneo in Malaysia, and a prominent hydroelectric plant was constructed. It is Malaysia's most extensive and tallest hydroelectric power station, with which the head dam is approximately 160 m and named Bakun Hydro-Electric Dam [3]. It has the highest power generation capacity of 2400MW in Sarawak. In addition, some other hydroelectric dams, such as Batang Ai (108MW) and Murum HEP (944MW), generate electricity for domestic usage. Meanwhile, many remote areas still do not have electricity accessibility [4]. To solve this problem, proper identification and integration of new locations of HES are necessary [5].

In this paper, all the potential HES are provided by SEB in a map. By geo-referencing the map using GIS spatial tool and pre-processing this data, it has been possible for this research to create reliable and extensive data output and place it on the Sarawak map. The longitude and latitude of these HES are retrieved. A total of 155 HES has been identified, utilized, and applied for AI approaches. Next, the paper aims to find the optimal transmission line routing for the identified HES with an improved hybrid AI approach. The key contributions of the research are listed as below.

• Provide the locations of potential HES in Sarawak State.
• Develop transmission line routing for identified HES in Sarawak State.
• Propose a robust hybrid AI algorithm for renewable site integration.
• A hybrid AI algorithm possesses parameter tuning features based on user preferences.

The paper is organized into the following sections. Section 1 presents the research introduction, and Section 2 reviews the past relevant research works critically. Then, Section 3 describes the methodology of the proposed hybrid AI approach. Section 4 shows the results and discussion, and Section 4.3 discusses the conclusion and future work.

## 2 Review study

Travelling Salesman Problem Method (TSPM) is a well-known method [6] to solve a sequence-based problem whereby the salesmen face difficulty in deciding the routing when they travel from a location to “*n*” number of locations [7]. TSP aims to determine the minimum total distance for overall trips. In this respect, many algorithms can be used for TSP applications. Simulated Annealing can solve TSP but is a single solution-based algorithm [8]; hence its solution can be easily trapped into the local optimum region compared to others. Particle Swarm Optimization (PSO) method is received researchers' attention nowadays as it performs better over many algorithms in terms of computational effort [9]. However, PSO depends highly on the best solution on population. It will easily cause the solution to fall into local optimum region [10][11]. The algorithms such as Genetic Algorithm (GA) [12] and Mixed-Integer Linear Programming (MILP) [13] are among the most well-known methods to solve the TSP scenario. These two methods can generate the optimal route for TSP but with different execution durations [14][15]. Conventional TSP-GA develops the optimal transmission routing with the following procedures [12][16]. At the initial state, the user input parameters will have to be set up [17]. These include the coordination data, distance matrix data, population size, number of iterations, and stopping criteria [18]. It is followed by constructing the fitness function of the TSP. Next, natural biological genetics processes such as selection, crossover, mutation, and reproduction are developed to produce the optimal routes [19]. Furthermore, proper consideration of mutation operator in GA can significantly reduce the risk of the solution falling into the local optimum region [15].

On the other hand, MILP is suitable to interact with the TSP complex optimization as well [20]. Fundamental input parameters of MILP are denoted [14] at first. Sequentially, the integer constant and number; inequality and equality matrices; inequality and equality vectors; lower boundary and upper boundary are defined. Next, the constraints of typical TSP scenarios are set to ensure the MILP technique that follows user-defined rules to search for the optimal solution in a feasible region [21][22]. In transmission line routing design, the application of the TSPM assists in searching the shortest path to integrate all the identified HES [23]. The ordinary TSPM optimizes the transmission line routing by relying on only the distance parameter [12]. Undeniably, this solution can reduce the costs of transmission lines as the total distance among HES is reduced [24]. However, more parameters can be added to comprehensively optimise the transmission line routing. It indicates that the conventional TSP-GA method is not powerful enough as it only manages to deal with the singular objective function [14]. In this regard, this paper presents an improved method that applies fuzzy TSP-GA to generate better transmission line routes depending on the parameters of distance and elevation difference.

As usual, the distance parameter is the primary concern when designing the transmission line routing as a shorter distance implies lower installation costs of transmission lines. For the additional parameter, the elevation difference between HES is considered. A higher elevation difference between the two HES would have a steeper base for installation of the transmission tower. The more vertical base increases the construction difficulty, which will cause a high installation cost. Therefore, the paper provides an improved hybrid method to generate the minimum total distance and minimum total elevation difference for identified HES. The only closest comparisons of researches found in the literature were summarized in Table 1. Currently, no research has been carried out so far using the combination of fuzzy TSP-GA. In addition, no hydro-based renewable energy research applications are utilized for the algorithms implemented and reported in this paper. Adding fuzzy logic to the algorithms makes this research superior in optimizing the transmission line routing and equally optimizing the distance and elevation difference parameters. Table 1 shows the summary of the literature studies.

**Table 1:** Summary of literature studies [5][6][8][9][10][13][16][17][18][19][21][22].

Author	Area	Method	Strength	Limitation
[5]	Optimization of Power Transmission Lines Routing	FAHP, GIS	Allow multi-inputs	Slow operation, lack of reliability, suitable for least number of routes only
[6]	Solving Large-scale TSP Problems	TSP-ACA	High robustness, high precision, simplicity	Slow operation, suitable to solve small and medium size of TSP problems only, suitable for singular TSP objective function only
[8]	Simulated Annealing in Maximizing the Thermal Conductance	SA	Simplicity, less restriction	Single-based solution
[9]	TSP Optimization using Genetic Algorithm	TSP-GA	High efficiency against complex problems	Suitable for singular TSP objective function only
[10]	XGBoost Optimized by Adaptive Particle Swarm Optimization for Credit Scoring	PSO	High efficiency,	High risk falling to local optimum region
[13]	Persistent Unmanned Aerial Vehicle Delivery Logistics	TSP-MILP	High efficiency, high flexibility	Not suitable for large-scale problems
[16]	Clustering for TSP Problems	Improved TSP-GA	High efficiency, able to handle large scale TSP problems with a shorter period	Suitable for singular TSP objective function only
[17]	Multiple TSP Problems	MTSP-PGA	High efficiency	Poor communication between individuals
[18]	Optimization with Fuzzy Control	FPSO-GA	High precision, search optimum results with high diversity	Complexity issue
[19]	Optimization of Carpool Service Problem	FGA	Short computation time, less complexity	Lack of flexibility
[21]	Cyber-attack on Overloading Multiple Lines	MILP	High efficiency, high Precision	Not suitable for large-scale problems, Suitable for singular TSP objective function only
[22]	Electrical Simulation Optimization Problems	MILP	High efficiency, short execution duration, high feasibility	Complexity issue

This research paper presented the improved hybrid AI algorithm to optimize the transmission line routes among 155 HES. The proposed AI algorithms are superior in integrating HES and work effectively in optimizing the multi-objective functions. Hence minimum values of total distance and elevation difference among 155 HES have been acquired. Furthermore, for the proposed algorithm architecture, fuzzy logic functions are developed to interact with multi-independent inputs, while the TSP-GA algorithm plays a crucial role in searching for the best transmission routing diversely from the global and local optimum regions.

## 3 Methodology

### 3.1 Application of spatial mapping by GIS tool

The identification of hydro energy sites is a very challenging task. The first task is to use spatial mapping since hydro energy potential is only available at the river locations throughout 1000 km from South to North or vice versa of Sarawak. Moreover, the terrain where rivers pass through has different land slope characteristics comprising all types of land conditions. The realization of spatial mapping is the best tool to deal with the land complex nature of diverse formations. The data gathered from these rivers in this vast land are divided into six districts as these are where the 155 HES located. Therefore, the coordinates of 155 HES are retrieved by geo-referencing the original map using the QGIS software tool. Each of them is labelled with the unique ID [26] as (1).

(1) $$\mathrm{HES}=[H_{1},H_{2},H_{3},H_{4},H_{5},\ldots ,H_{155}]$$

The process of HES integration starts after all their coordinates are identified.

### 3.2 Genetic algorithm

Generally, TSP-GA reflects the natural selection process that applies biological evolution theory. The addition of GA to the TSP scenario possesses five major operations as (A), (B), (C), (D) and (E). The execution of the TSP-GA algorithm is customarily performed using MATLAB software. Hence, the best transmission routing among HES can be generated, and it depends on the distance parameter only.

*A)* *Declaration of Inputs:*

$$\begin{array}{cc} \mathrm{XY}:\; & \text{Coordination of HES locations}\\ D_{m}:\; & \text{Distance matrix of HES locations}\\ P_{s}:\; & \text{Size of population}\\ I_{n}:\; & \text{Number of iterations}\\ S_{p}:\; & \text{Show GA's progress if true}\\ S_{R}:\; & \text{Show GA's results if true}\\ S_{\mathrm{wb}}:\; & \text{Show GA's wait-bar if true} \end{array}$$

*B)* *Formation of Fitness Function:*

TSP-GA is used to determine the minimum value of the objective function. The objective function [16] is expressed as (2).

(2) $$\min f\left(x\right)=\sum_{i=1}^{j}d({\mathrm{HES}}_{i},{\mathrm{HES}}_{i+1})+d({\mathrm{HES}}_{n},{\mathrm{HES}}_{1})$$

The fitness function is defined as the inversion of the developed objective function. It is to assure the lower the minimum total distance, the higher the fitness value. Equation (3) [16] shows the fitness function of the GA approach. Thus, the individual with a higher fitness value is set to reproduce, while the individual with a low fitness value will be eliminated.

(3) $$f\left(i\right)=\frac{1}{\min d_{t}\sum_{i=1}^{j}d({\mathrm{HES}}_{i},{\mathrm{HES}}_{i+1})+d({\mathrm{HES}}_{n},{\mathrm{HES}}_{1})}$$

*C)* *Selection Operator:*

The best two individuals are selected according to the competition. The first winning individual, $I_{1}$ must have the highest fitness value, max⁡f(i). Besides, the second winning individual is selected using a roulette wheel. To perform this, the probability of each individual is identified [16] as (4).

(4) $$fP_{i}=\frac{f(i)}{\sum_{i=1}^{P_{s}}f(i)}$$

This is followed by determining the cumulative probability of each individual [16] as (5).

(5) $$Q_{i}=\sum_{j=1}^{i}P_{j}$$

Now roulette wheel must generate a random number in the range [16] as (6),

(6) $$\min\left(Q_{i}\right)\leq \mathrm{rand}\leq \max(Q_{i})$$

Next, the winner of the second individual is determined [16] by (7),

(7) $$\text{If}\;Q_{i}\leq \mathrm{rand}\leq Q_{i+1},\;\text{select}\;Q_{i+1}$$

*D)* *Crossover Operator:*

Both winning individuals, $I_{1}$ and $I_{2}$ are considered as the parents. Recombination of the parental genes is required to produce four better offsprings. The crossover mechanism is necessary to enhance searchability and meet the optimal solution. The rules of gene recombination are set as shown in Table 2. $R_{\mathrm{os}}$ is the first departing HES site that refers to the first gene in an offspring, while $R_{\mathrm{oa}}$ is the next nearby HES site that refers to the second gene. In the beginning, $R_{\mathrm{os}}$ in the new offspring has to follow $R_{\mathrm{os}}$ in either $I_{1}$ or $I_{2}$. Then, $R_{\mathrm{os}}$ will connect to $R_{\mathrm{oa}}$ based on selection criteria (9). Sequentially, $R_{\mathrm{oa}}$ becomes the new $R_{\mathrm{os}}$ and it connects to new $R_{\mathrm{oa}}$. The process will loop until the termination condition, *t* is met (10). Four unique offsprings (O_1_, O_2_, O_3_, O_4_) are formed at the end. Among them, the offspring with the minimum total distance of the transmission routing will be selected as the winner.

**Table 2:** Rules of parental genes recombination for four offsprings.

Offspring	Departing RES site, *R*_*os*_	Adjacent RES site, *R*_*oa*_
O_1_	*I*_1_	Forward direction; selection between *I*_1_ and *I*_2_
O_2_	*I*_1_	Backward direction; selection between *I*_1_ and *I*_2_
O_3_	*I*_2_	Forward direction; selection between *I*_1_ and *I*_2_
O_4_	*I*_2_	Backward direction; selection between *I*_1_ and *I*_2_

Two types of distances, $d_{x}$ and $d_{y}$ are defined [17] as (8):

(8) $$d_{x}=\text{distance between }R_{\mathrm{os}}\text{ and }R_{\mathrm{oa}}\text{ in }I_{1}\;d_{y}=\text{distance between }R_{\mathrm{os}}\text{ and }R_{\mathrm{oa}}\text{ in }I_{2}$$

Crossover Selection Criteria is fulfilled [17] by (9) and (10):

(9) $$\text{If }d_{x}<d_{y},\text{ then select }R_{\mathrm{oa}}\text{ with }d_{x},\text{ otherwise }R_{\mathrm{oa}}\text{ with }d_{y}.\text{If only }d_{x}\text{ exists},\text{ then select }R_{\mathrm{oa}}\text{ with }d_{x},\text{ and vice versa}.$$

(10) $$t:\text{Number of new}\;R_{\mathrm{oa}}=0$$

*E)* *Mutation Process:*

The selected offspring is then mutated to produce three new offsprings. It is performed by randomising the order of genes in the selected offspring. The main reason of considering mutation process is to reduce the risk of the solution falling into the local optimum region. Besides, it improves the algorithm diversity to explore the global search space further. The three types of random resetting techniques are demonstrated as follows:

• Swap mutation: Choose any two HES and interchange their order.
• Scramble mutation: Choose any subset (half of the total HES) and scramble their order.
• Inversion mutation: Select any subset (half of the total HES) offspring and invert their order.

The last winning offspring must comprise transmission routing with minimum total distance. It is selected from the original offspring and three mutated offsprings (swap, scramble, and inversion).

### 3.3 Fuzzy operation

In solving the standard TSP scenario, only the distance criterion is considered. Thus, the optimal transmission line route is obtained when the minimum value of total distance is successfully found. The fuzzy operation is applied to the existing TSP-GA to acquire more comprehensive results since it can deal with multi-objective functions. The second criterion, elevation difference is included in the design of the transmission line routing. Thus, the complete multi-objective function [18] is as shown in (11).

(11) $$\min\;f\left(x\right)=\left\{ \begin{array}{c} \sum_{i=1}^{j}d({\mathrm{HES}}_{i},{\mathrm{HES}}_{i+1})+d({\mathrm{HES}}_{n},{\mathrm{HES}}_{1})\\ \sum_{i=1}^{j}\Delta e({\mathrm{HES}}_{i},{\mathrm{HES}}_{i+1})+\Delta e({\mathrm{HES}}_{n},{\mathrm{HES}}_{1}) \end{array}\right.$$

**Figure 1:**
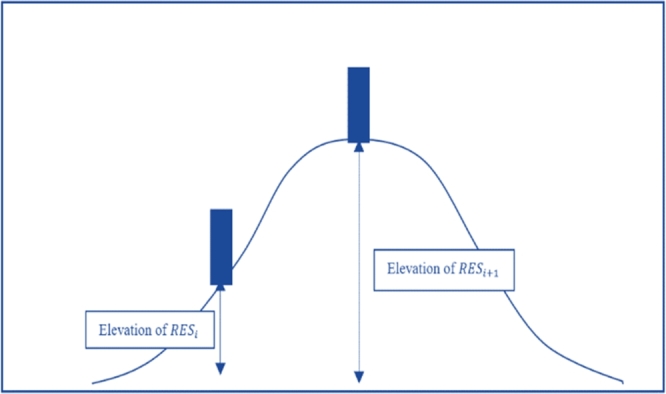
Elevation difference between 2 HES [26].

Elevation difference, Δ*e* is added to the optimization process due to its significance. When the elevation difference of the two HES is high, it indicates the slope between those two HES is steeper. Therefore, it increases the construction difficulty and leads to higher installation costs of HES and transmission line towers. Fig. 1 depicts the elevation difference of any two HES.

Elevation difference can be determined [26] by (12):

(12) $$\text{Elevation difference},\;\Delta e=\left|{\mathrm{RES}}_{i+1}-{\mathrm{RES}}_{i}\right|$$

To generate Δ*e* in matrix data format [26] with MATLAB, using (13):

(13) $$\Delta e=\text{squareform}[\text{pdist}(d_{s})]$$

Where $d_{s}$ = participated district.

When dealing with multi-objective functions that contain distance and elevation difference variables, algorithm hybridization is recommended to generate the optimal solution. Therefore, hybridization of fuzzy operation and TSP-GA approach is proposed to solve the encountered problem. At first, all the 155 HES locations are retrieved by geo-referencing the potential HES map provided by SEB. After identifying their coordinates, all HES are sorted into 6 districts. Hence, each district will have a set number of HES. Then, the distance, *d* matrix data and elevation difference, Δ*e* matrix data are constructed for all 6 districts. The matrix data formats of *d* and Δ*e* are as shown in Table 3(a), Table 3(b) respectively.

**Table 3(a):** Matrix data format of *d* data [26].

*d*	*H*_1_	*H*_2_	*H*_3_	⋯	*H*_*n*_
*H*_1_	$d_{H_{1}H_{1}}$	$d_{H_{1}H_{2}}$	$d_{H_{1}H_{3}}$	⋯	$d_{H_{1}H_{n}}$
*H*_2_	$d_{H_{2}H_{1}}$	$d_{H_{2}H_{2}}$	$d_{H_{2}H_{3}}$	⋯	$d_{H_{2}H_{n}}$
*H*_3_	$d_{H_{3}H_{1}}$	$d_{H_{3}H_{2}}$	$d_{H_{3}H_{3}}$	⋯	$d_{H_{3}H_{n}}$
⋯	⋯	⋯	⋯	⋯	⋯
*H*_*n*_	$d_{H_{n}H_{1}}$	$d_{H_{n}H_{2}}$	$d_{H_{n}H_{3}}$	⋯	$d_{H_{n}H_{n}}$

**Table 3(b):** Matrix data format of Δ*e* data [26].

Δ*e*	*H*_1_	*H*_2_	*H*_3_	⋯	*H*_*n*_
*H*_1_	$\Delta e_{H_{1}H_{1}}$	$\Delta e_{H_{1}H_{2}}$	$\Delta e_{H_{1}H_{3}}$	⋯	$\Delta e_{H_{1}H_{n}}$
*H*_2_	$\Delta e_{H_{2}H_{1}}$	$\Delta e_{H_{2}H_{2}}$	$\Delta e_{H_{2}H_{3}}$	⋯	$\Delta e_{H_{2}H_{n}}$
*H*_3_	$\Delta e_{H_{3}H_{1}}$	$\Delta e_{H_{3}H_{2}}$	$\Delta e_{H_{3}H_{3}}$	⋯	$\Delta e_{H_{3}H_{n}}$
⋯	⋯	⋯	⋯	⋯	⋯
*H*_*n*_	$\Delta e_{H_{n}H_{1}}$	$\Delta e_{H_{n}H_{2}}$	$\Delta e_{H_{n}H_{3}}$	⋯	$\Delta e_{H_{n}H_{n}}$

$H_{1},\;H_{2}$ and $H_{3}$ are the HES; $H_{n}$ refers to last HES. The distance, *d* matrix data and elevation difference, Δ*e* matrix data are retrieved in QGIS tool as the input data. After that, the fuzzy logic approach integrates both *d* and Δ*e* matrix data to produce more reliable outputs. In the fuzzy MATLAB toolbox, it possesses the four main sections as follows:

*A)* *Inputs and Output Definitions:*

The inputs of distance, *d* and elevation difference, Δ*e* are set. Meanwhile, penalty distance, $p_{d}$ is set as the output.

*B)* *Membership Functions Development:*

All inputs and output have 5 triangular membership functions. The triangular membership function [26] is formulated as:

(14) $$\mu_{A}\left(x:a,b,c,\right)=\left\{ \begin{array}{cc} 0,\; & x\leq a\\ \frac{x-a}{b-a},\; & a\leq x\leq b\\ \frac{c-x}{c-b},\; & b\leq x\leq c\\ 0,\; & c\leq x \end{array}\right.$$

Each input and output are attached with five membership functions:

• Input *d*: Very Short (V_S_), Short (S), Medium (M), Long (L), and Very Long (V_L_)
• Input Δ*e*: Very Low (V_L_), Short (L), Medium (M), High (H), and Very High (V_H_)
• Output $p_{d}$: Very Short (V_S_), Short (S), Medium (M), Long (L), and Very Long (V_L_)

**Figure 2:**
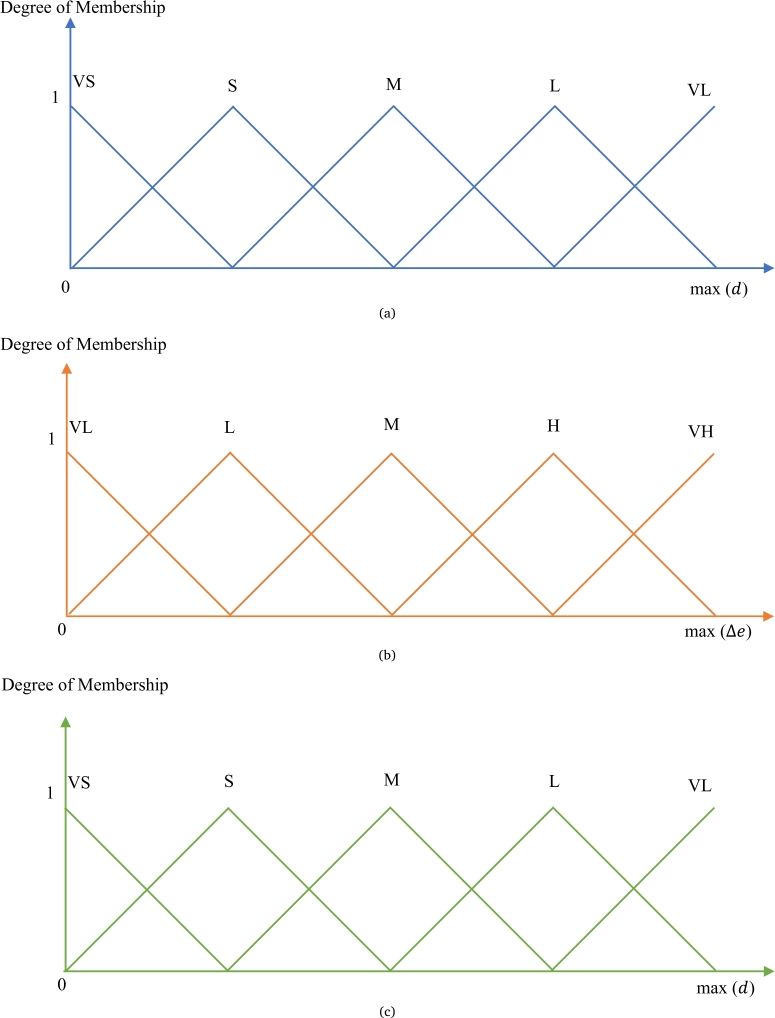
(a) Triangular membership function for input *d*[26]. (b) Triangular membership function for input Δ*e*[26]. (c) Triangular membership function for output *p*_*d*_[26].

Four common membership functions named triangular, trapezoidal, sigmoid and gaussian. Triangular membership is chosen due to its simplicity and flexibility. Mamdani Fuzzy Inference System (FIS) is utilized as the control system due to its interpretability. For the membership functions of each input and output, the minimum value is set as 0. The maximum value of the input *d* is set as the maximum value of matrix data *d*; the maximum value of the input Δ*e* is set as the maximum value of matrix data Δ*e*. The maximum value of output $p_{d}$ is set as the maximum value of matrix data *d* as well. Figs. 2(a), 2(b), and 2(c) depict the membership function graphs for *d*, Δ*e* and $p_{d}$ respectively.

As the minimum and maximum values are determined for each input and output, every membership function consists of four regular intervals [18] as equation (15).

(15) $$1^{\mathrm{st}}\;\text{interval}=[\frac{1}{4}\max\left(d\right)]2^{\mathrm{nd}}\;\text{interval}=[\frac{1}{4}\max\left(d\right),\frac{1}{2}\max\left(d\right)]3^{\mathrm{rd}}\;\text{interval}=[\frac{1}{2}\max\left(d\right),\frac{3}{4}\max\left(d\right)]4^{\mathrm{th}}\;\text{interval}=[\frac{3}{4}\max\left(d\right),\max\left(d\right)]$$

*C)* *Setting Fuzzy Rules:*

Table 4 demonstrates the two inputs are assumed to be equally important to each other. The first left column refers to the input *d*, while the first upper row refers to input Δ*e*. The other data in Table 4 represents output $p_{d}$.

**Table 4:** Fuzzy rules in FIS [26].

Distance∖Elevation	Very Low	Low	Moderate	High	Very High
Very Short	Very Short	Very Short	Short	Short	Medium
Short	Very Short	Short	Short	Medium	Long
Medium	Short	Short	Medium	Long	Long
Long	Short	Medium	Long	Long	Very Long
Very Long	Medium	Long	Long	Very Long	Very Long

These fuzzy rules are set in a Fuzzy Inference System (FIS). The 25 fuzzy rules have been created. All expressions of fuzzy rules [26] are shown in equation (16).

(16) $$\begin{array}{ccc} {}[d=\text{Very Short},\; & \Delta e=\text{Very Low}]\; & \to [p_{d}=\text{Very Short}]\\ {}[d=\text{Very Short},\; & \Delta e=\text{Low}]\; & \to [p_{d}=\text{Very Short}]\\ {}[d=\text{Very Short},\; & \Delta e=\text{Moderate}]\; & \to [p_{d}=\text{Short}]\\ {}[d=\text{Very Short},\; & \Delta e=\text{High}]\; & \to [p_{d}=\text{Short}]\\ {}[d=\text{Very Short},\; & \Delta e=\text{Very High}]\; & \to [p_{d}=\text{Medium}]\\ {}[d=\text{Short},\; & \Delta e=\text{Very Low}]\; & \to [p_{d}=\text{Very Short}]\\ {}[d=\text{Short},\; & \Delta e=\text{Low}]\; & \to [p_{d}=\text{Short}]\\ {}[d=\text{Short},\; & \Delta e=\text{Moderate}]\; & \to [p_{d}=\text{Short}]\\ {}[d=\text{Short},\; & \Delta e=\text{High}]\; & \to [p_{d}=\text{Medium}]\\ {}[d=\text{Short},\; & \Delta e=\text{Very High}]\; & \to [p_{d}=\text{Long}]\\ {}[d=\text{Medium},\; & \Delta e=\text{Very Low}]\; & \to [p_{d}=\text{Short}]\\ {}[d=\text{Medium},\; & \Delta e=\text{Low}]\; & \to [p_{d}=\text{Short}]\\ {}[d=\text{Medium},\; & \Delta e=\text{Moderate}]\; & \to [p_{d}=\text{Medium}]\\ {}[d=\text{Medium},\; & \Delta e=\text{High}]\; & \to [p_{d}=\text{Long}]\\ {}[d=\text{Medium},\; & \Delta e=\text{Very High}]\; & \to [p_{d}=\text{Long}]\\ {}[d=\text{Long},\; & \Delta e=\text{Very Low}]\; & \to [p_{d}=\text{Short}]\\ {}[d=\text{Long},\; & \Delta e=\text{Low}]\; & \to [p_{d}=\text{Medium}]\\ {}[d=\text{Long},\; & \Delta e=\text{Moderate}]\; & \to [p_{d}=\text{Long}]\\ {}[d=\text{Long},\; & \Delta e=\text{High}]\; & \to [p_{d}=\text{Long}]\\ {}[d=\text{Long},\; & \Delta e=\text{Very High}]\; & \to [p_{d}=\text{Very Long}]\\ {}[d=\text{Very Long},\; & \Delta e=\text{Very Low}]\; & \to [p_{d}=\text{Medium}]\\ {}[d=\text{Very Long},\; & \Delta e=\text{Low}]\; & \to [p_{d}=\text{Long}]\\ {}[d=\text{Very Long},\; & \Delta e=\text{Moderate}]\; & \to [p_{d}=\text{Long}]\\ {}[d=\text{Very Long},\; & \Delta e=\text{High}]\; & \to [p_{d}=\text{Very Long}]\\ {}[d=\text{Very Long},\; & \Delta e=\text{Very High}]\; & \to [p_{d}=\text{Very Long}] \end{array}$$

*D)* *Generate Penalty Distance*, $p_{d}$*Matrix Data:*

**Figure 3:**
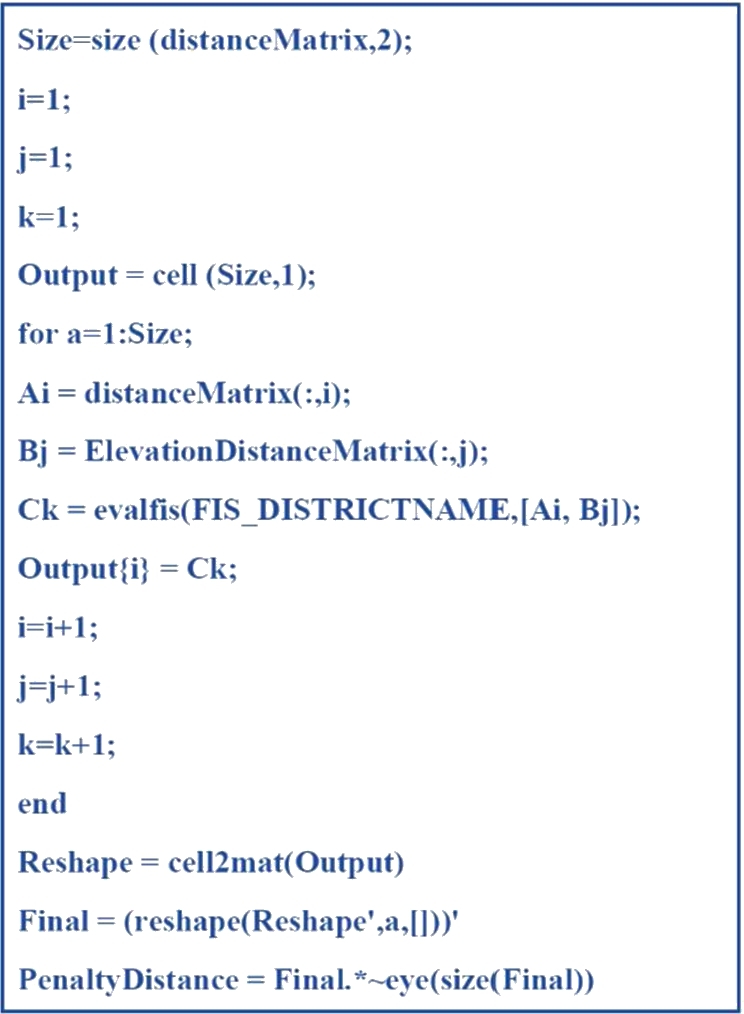
Production of *p*_*d*_ matrix data using MATLAB [26].

At this point, users can alter the inputs based on their preferences. As fuzzy logic operation manages to overcome vague environment, hence optimal solution can be achieved by tuning two independent inputs. Besides, when both inputs matrix data are added to the FIS, a new set of $p_{d}$ matrix data is acquired. Fig. 3 shows the MATLAB coding for $p_{d}$ matrix data generation.

*E)* *Produce Virtual Distance*, $V_{d}$*matrix data:*

The $V_{d}$ matrix data [26] can be obtained using (17):

(17) $$V_{d}\text{ matrix data}=d\text{ matrix data}+p_{d}\text{ matrix data}$$

By adding the original *d* matrix data to $p_{d}$ matrix data, $V_{d}$ matrix data is formed. The $V_{d}$ matrix data is influenced by input *d* and input Δ*e*. After this, the new set of $V_{d}$ matrix data can be computed using the TSP-GA technique to produce the optimal transmission routes.

## 4 Results and discussion

### 4.1 Selection of HES

**Figure 4:**
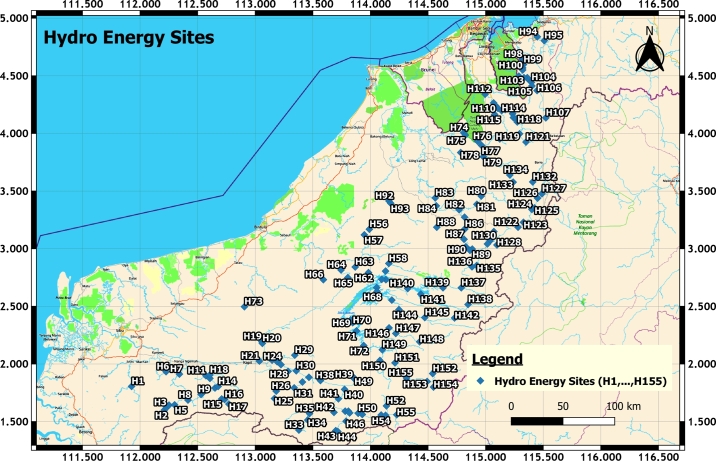
Potential HES in Sarawak State [25].

This section discussed the results of the selection and integration of HES. Determining HES is challenging as only locations with river flow could be selected. Fortunately, SEB has the raw data mapping of potential HES for the whole Sarawak. The geo-referencing method in GIS can retrieve the coordinates of all HES. At last, there are 155 HES are identified and selected. Meanwhile, their latitude and longitude are obtained for the next phase of HES integration. Fig. 4 depicts all 155 potential HES in Sarawak. Each of them is labelled sequentially from H1 to H155.

**Figure 5:**
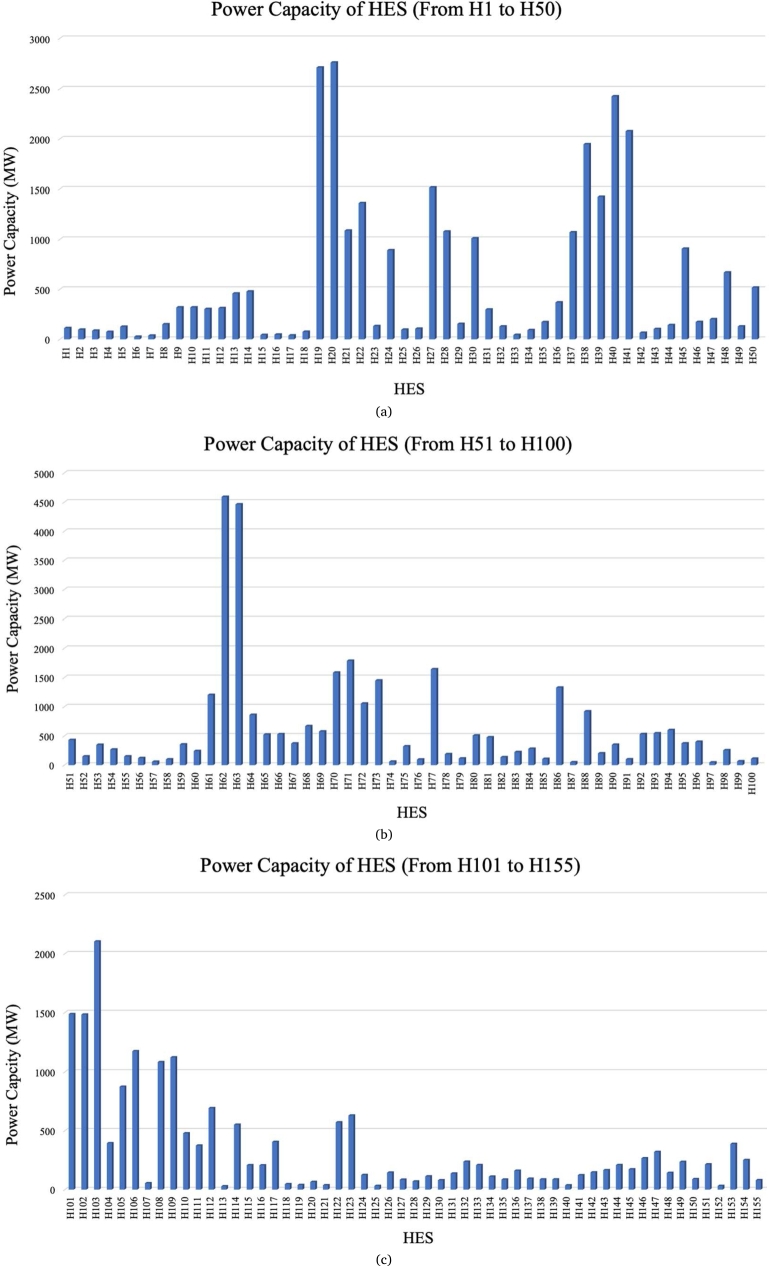
(a) Power capacity of HES (from H1 to H50) [26]. (b) Power capacity of HES (from H51 to H100) [26]. (c) Power capacity of HES (from H101 to H155) [26].

Apart from that, the power capacity of the 155 HES was also retrieved from the spatial mapping. Thus, Figs. 5(a), 5(b) and 5(c) depict the power capacity of 155 HES.

### 4.2 Integration of HES using fuzzy based TSP-GA

**Figure 6:**
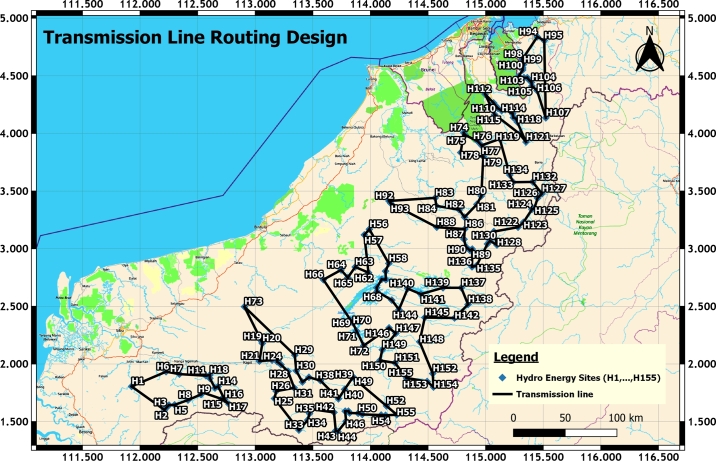
Transmission routing design in TSP-GA or TSP-MILP [26].

All 155 HES are initially divided into 6 districts: Song, Kapit, Belaga, Marudi, Limbang and Lawas District. From this, six ring topologies are produced. The Song District consists of 18 HES; Kapit District possesses 38 HES; Belaga comprises 36 HES; Marudi has 36 HES; Limbang owns 13 HES; Lawas occupies 14 HES. In the first analysis, TSP-GA is utilized to generate the transmission routing for HES for finding $d_{t}$ values for all the HES in 6 districts. Another technique of TSP-Mixed Integer Linear Programming [27] is adopted to validate the $d_{t}$ results in TSP-GA. Since both TSP-GA and TSP-MILP generated identical $d_{t}$ values and have exactly the same transmission routing configuration as depicted in Fig. 6, the minimum total distance results are validated. Form this, there is only one alternative algorithm, TSP-MILP is utilized for validation because both results in TSP-GA and TSP-MILP converge very fast toward to optimal solution. Thus, it is concluded that when only the distance parameter is considered for transmission routing design for these grouped 155 HES, the result presented in Fig. 6 is the optimal solution.

**Figure 7:**
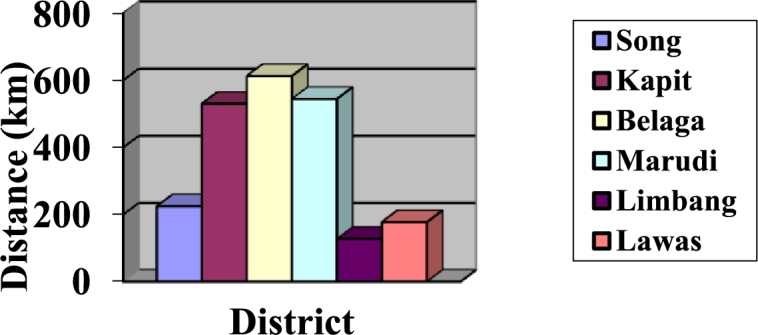
*d*_*t*_ of 6 districts using TSP-GA [26].

The distance and elevation difference of HES in 6 districts using TSP-GA are illustrated in Appendix A. The minimum total distances, $d_{t}$ for all six ring topologies have been determined using TSP-GA. Table 5 and Fig. 7 highlight the $d_{t}$ values in km.

**Table 5:** *d*_*t*_ of HES integration using TSP-GA [26].

District	*d*_*t*_ (km)
Song	224.50
Kapit	530.08
Belaga	612.74
Marudi	544.80
Limbang	127.18
Lawas	177.09

**Figure 8:**
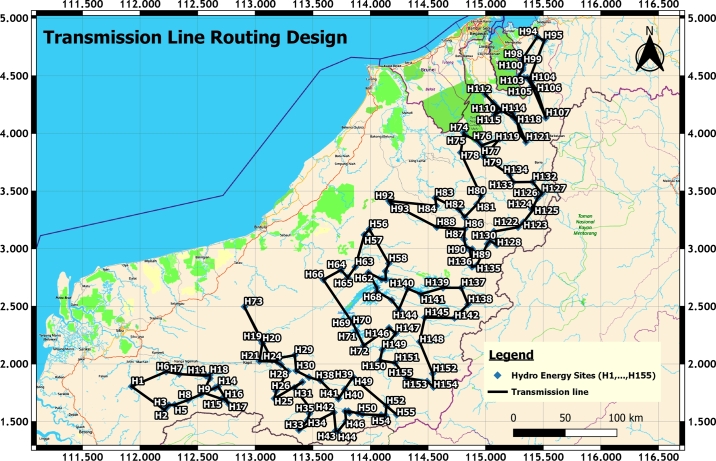
Transmission Routing Design in Fuzzy TSP-GA [26].

The fuzzy TSP-GA is designed to achieve the minimum values of total distance, $d_{t}$ and total elevation difference, $\Delta e_{t}$, thus, these two parameters are taken into account in results validation. The $\Delta e_{t}$ in TSP-GA for each district is generated using GIS by referring to its transmission routing. Fig. 8 depicts the results of transmission routing using fuzzy TSP-GA.

**Figure 9:**
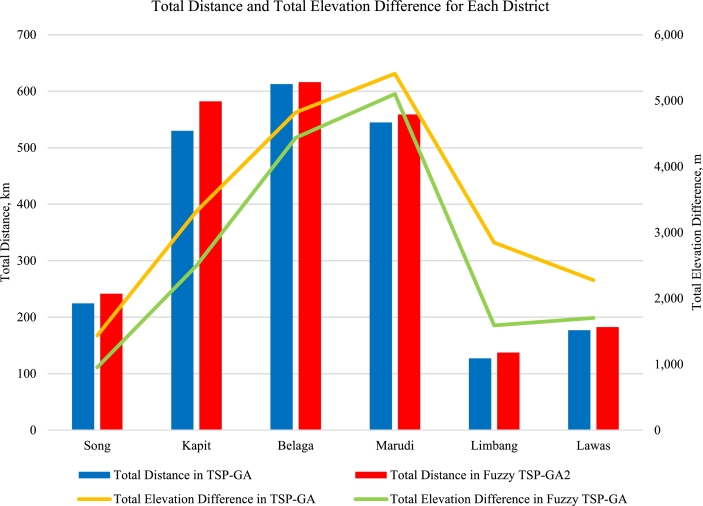
*d*_*t*_ (km) and Δ*e*_*t*_ (m) of Each District using TSP-GA and Improved Fuzzy TSP-GA [26].

If only distance parameter is considered for routing design, the intersection of transmission lines seldom appears. Since fuzzy TSP-GA interacts with two objective functions, the transmission routing pattern will be considerably diverse. The $d_{t}$ and $\Delta e_{t}$ parameters in TSP-GA are assumed to have the same margin percentages at 50%. This implies that both $d_{t}$ and $\Delta e_{t}$ are equally significant parameters. The net change percentages of $d_{t}$ and $\Delta e_{t}$ in fuzzy TSP-GA are calculated based on the margin percentages in TSP-GA. Next, the percentages of $d_{t}$ and $\Delta e_{t}$ in TSP-GA and fuzzy TSP-GA are added respectively. As fuzzy TSP-GA aims to obtain the minimum $d_{t}$ and $\Delta e_{t}$ values for all six districts, its transmission routing is further improved if the total percentage of $d_{t}$ and $\Delta e_{t}$ is less than 100%. Table 6 and Fig. 9 indicate the total distance, $d_{t}$ and total elevation difference, $\Delta e_{t}$ values in TSP-GA and fuzzy TSP-GA; improved factor, $f_{i}\left(\text{\%}\right)$ [26] is as equation (18) and the distance and elevation difference of HES in 6 districts using Fuzzy TSP-GA are demonstrated in Appendix A.

**Table 6:** *d*_*t*_ and Δ*e*_*t*_ of each district using TSP-GA and improved fuzzy TSP-GA [26].

District	Algorithm	$d_{t}$		$\Delta e_{t}$		*d*_*t*_ % + Δ*e*_*t*_ %
		(km)	%	(m)	%	
Song	TSP-GA	224.50	50.00	1,434	50.00	100.00
	Fuzzy TSP-GA	241.63	53.81	952	33.19	87.01

Kapit	TSP-GA	530.08	50.00	3,322	50.00	100.00
	Fuzzy TSP-GA	582.18	54.91	2,496	37.57	92.48

Belaga	TSP-GA	612.74	50.00	4,822	50.00	100.00
	Fuzzy TSP-GA	616.06	50.27	4,438	46.02	96.29

Marudi	TSP-GA	544.80	50.00	5,410	50.00	100.00
	Fuzzy TSP-GA	559.01	51.30	5,102	47.15	98.46

Limbang	TSP-GA	127.18	50.00	2,846	50.00	100.00
	Fuzzy TSP-GA	137.50	54.06	1,590	27.93	81.99

Lawas	TSP-GA	177.09	50.00	2,276	50.00	100.00
	Fuzzy TSP-GA	182.63	51.56	1,704	37.43	89.00

(18) $$f_{i}\left(\text{\%}\right)=100\text{\%}-{\text{Fuzzy}}_{\mathrm{TSP}.\mathrm{GA}}\left[d_{t}\left(\text{\%}\right)+\Delta e_{t}\left(\text{\%}\right)\right]$$

**Figure 10:**
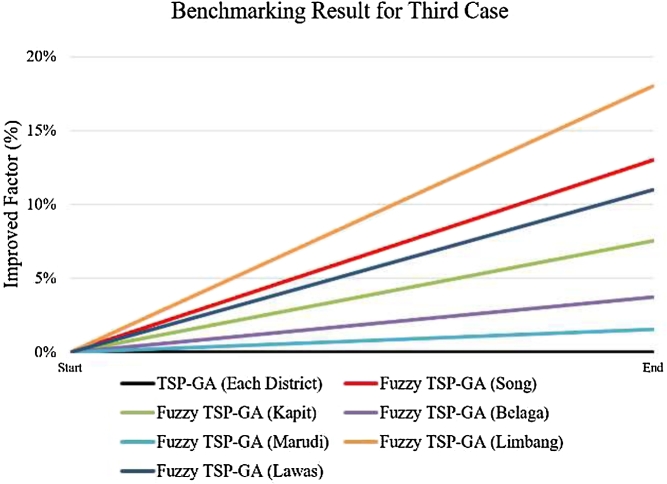
Benchmarking Results for Fuzzy TSP-GA and TSP-GA [26].

The $f_{i}\left(\text{\%}\right)$ represented the algorithm efficiency to meet the minimum values for both $d_{t}$ and $\Delta e_{t}$ parameters. Each district in TSP-GA has $f_{i}\left(\text{\%}\right)=0$. The result is improved if the district has a positive percentage, and the result deteriorates if the district has a negative percentage. The benchmarking tests revealed that all 6 districts have results improvements using fuzzy TSP-GA. Fuzzy TSP-GA successfully improved 12.99% for Song district, 7.52% for Kapit district, 3.71% for Belaga district, 1.54% for Marudi district, 18.01% for Limbang district, 11.00% for Lawas district compared to TSP-GA approach. Fig. 10 and Table 7 show the fuzzy TSP-GA method successfully further optimized the results in TSP-GA.

**Table 7:** $f_{i}\left(\text{\%}\right)$ in improved fuzzy TSP-GA algorithms [26].

Algorithm	TSP-GA	Fuzzy TSP-GA					
District	Each	Song	Kapit	Belaga	Marudi	Limbang	Lawas
$f_{i}\;\left(\text{\%}\right)$	0.00	12.99	7.52	3.71	1.54	18.01	11.00

Table 7 shows the improved factor for the improved fuzzy TSP-GA algorithm.

The solution time, $t_{s}$ for each district in TSP-GA and fuzzy TSP-GA are gathered using the tic−toc function in MATLAB and demonstrated in Table 8. It is necessary to determine the time solution for each district to measure the computational effort. Based on Table 8, the solution time for all the districts in TSP-GA and fuzzy TSP-GA is less than 10 seconds. The results of time solution are reasonable as 155 HES were divided into 6 districts, and each district has a low number of HES only (less than 40 HES). Hence, it drastically reduces the difficulty of GA searching the optimal results.

**Table 8:** $f_{i}\left(\text{\%}\right)$ time solution for each district in TSP-GA and fuzzy TSP-GA [26].

District	Algorithm	Solution Time, $t_{s}$ (s)
Song	TSP-GA	6.592
	Fuzzy TSP-GA	6.637

Kapit	TSP-GA	9.342
	Fuzzy TSP-GA	9.491

Belaga	TSP-GA	8.388
	Fuzzy TSP-GA	8.240

Marudi	TSP-GA	8.645
	Fuzzy TSP-GA	8.921

Limbang	TSP-GA	4.649
	Fuzzy TSP-GA	4.733

Lawas	TSP-GA	4.528
	Fuzzy TSP-GA	4.489

### 4.3 Conclusion and future work

In these recent years, the escalation of energy demand in Sarawak has endorsed in exploration of new renewable energy sites. In this paper, 155 HES were successfully identified in the interior state of Sarawak. These HES have been fully analyzed thoroughly in terms of mathematical modelling, using spatial GIS and AI algorithms detailed in the paper. This research has intensively focused on locating HES, grouping HES in their proximity wise, mapping and optimizing their distances and elevations, and aligning them into the transmission line routing. As detailed in Section 4, all 155 HES were identified, grouped into 6 districts, and utilized the powerful hybrid AI algorithms comprehensively to produce optimal solutions in transmission line routing. It has been proven that the integration of HES is improved and optimized using the proposed hybrid AI approaches. The presented results have demonstrated the capability of fuzzy in handling the multiple objective functions as one key achievement of this research paper. Also, fuzzy logic operation in the system allows user to tune the involved parameters according to user preferences. The distance and elevation difference are the critical parameters involved in transmission line routing design. Conventional TPS-GA is utilized to compare its results with the improved fuzzy TSP-GA's results to validate the proposed algorithm's efficiency. The benchmarking results highlight fuzzy TSP-GA successfully improved 12.99% for Song district, 7.52% for Kapit district, 3.71% for Belaga district, 1.54% for Marudi district, 18.01% for Limbang district, 11.00% for Lawas district against the TSP-GA. This work can be extended by considering more influential parameters such as lightning incidence to achieve a more complete result.

## Declarations

### Author contribution statement

F. Chen Jong: Conceived and designed the experiments; Performed the experiments; Analyzed and interpreted the data; Contributed reagents, materials, analysis tools or data; Wrote the paper.

Musse Mohamud Ahmed: Performed the experiments; Analyzed and interpreted the data; Contributed reagents, materials, analysis tools or data; Wrote the paper.

W. Kin Lau: Contributed reagents, materials, analysis tools or data.

H. Aik Denis Lee: Analyzed and interpreted the data; Contributed reagents, materials, analysis tools or data.

### Funding statement

This research work is financially supported by Vice Chancellor High Impact Research Grant [UNI/F02/VC-HIRG/85514/P11-03/2022] and Sarawak Digital Economy Corporation (SDEC) [RG/F02/SMA/10/2018]. Many thanks also forward to the researchers from the Department of Electrical and Electronic Engineering (UNIMAS) and Sarawak Energy Berhad (SEB).

### Data availability statement

Data will be made available on request

### Declaration of interests statement

The authors declare no conflict of interest.

### Additional information

No additional information is available for this paper.

## Figures and Tables

**Figure:**
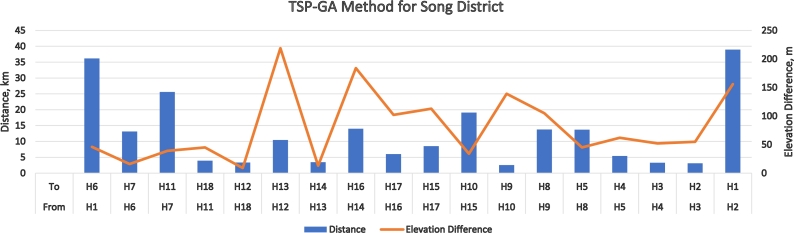


**Figure:**
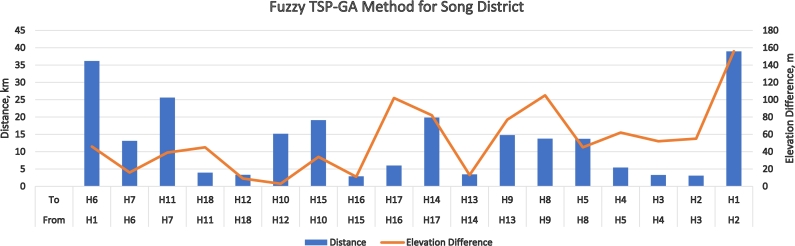


**Figure:**
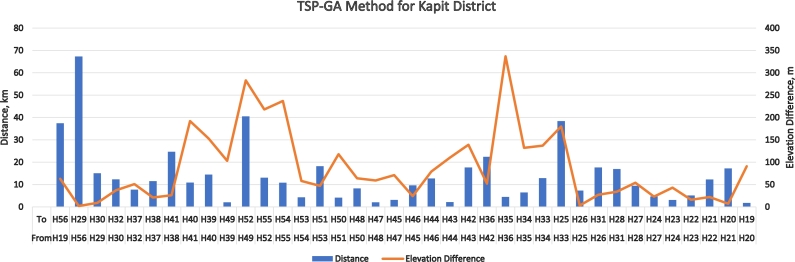


**Figure:**
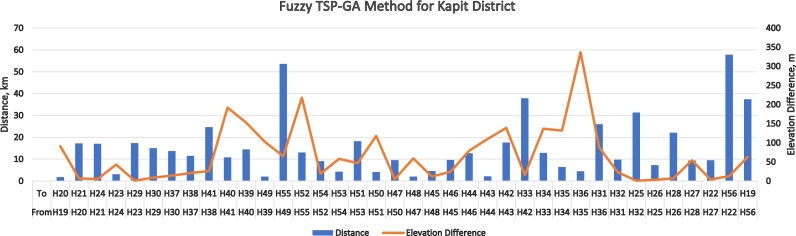


**Figure:**
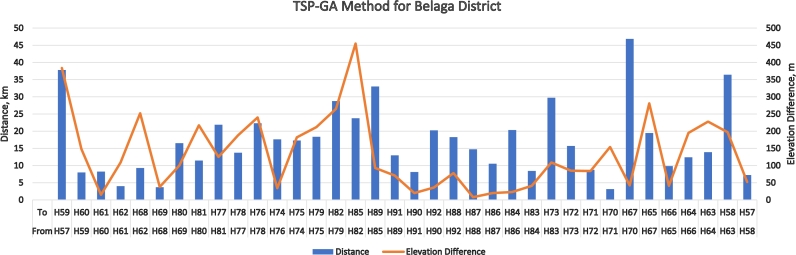


**Figure:**
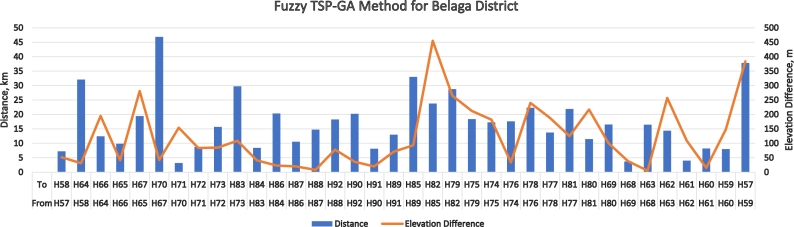


**Figure:**
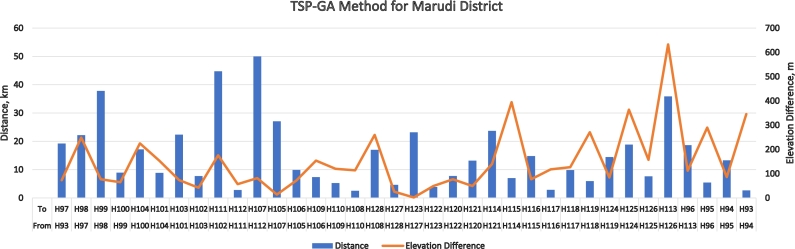


**Figure:**
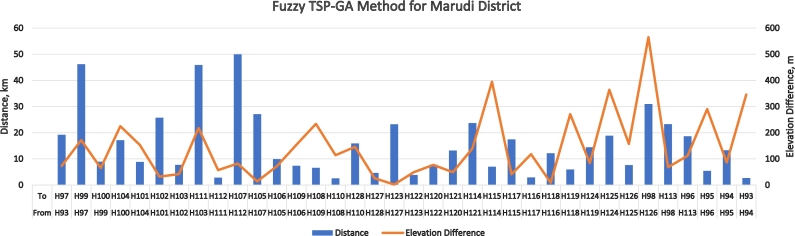


**Figure:**
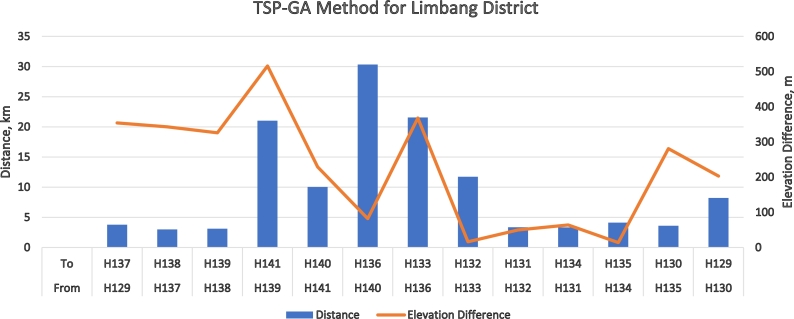


**Figure:**
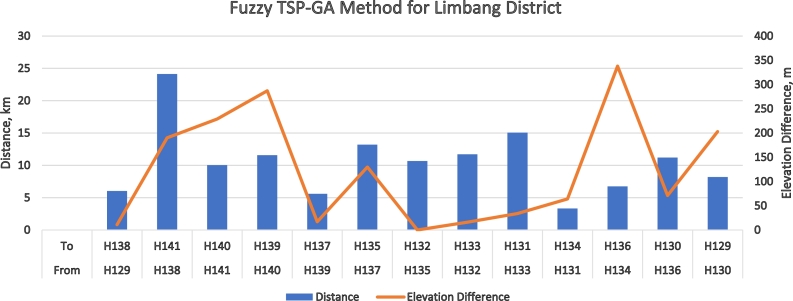


**Figure:**
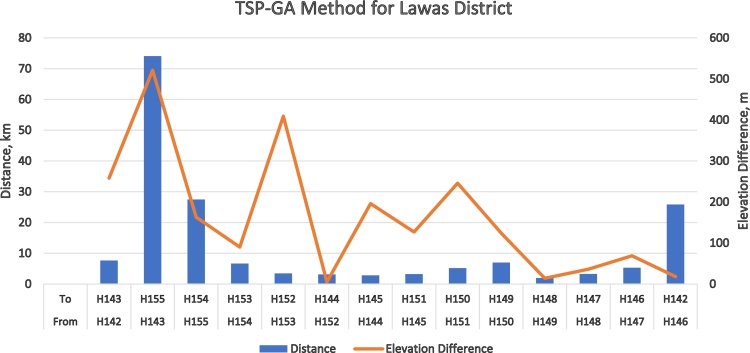


**Figure:**